# The effectiveness of multidisciplinary care models for patients with chronic kidney disease: a systematic review and meta-analysis

**DOI:** 10.1007/s11255-017-1679-7

**Published:** 2017-08-30

**Authors:** Yu Shi, Jiachuan Xiong, Yan Chen, Junna Deng, Hongmei Peng, Jinghong Zhao, Jing He

**Affiliations:** Institute of Nephrology of Chongqing and Kidney Center of PLA, Xinqiao Hospital, Third Military Medical University, Chongqing, China

**Keywords:** Chronic kidney disease, Multidisciplinary, Care, Systematic reviews and meta-analysis

## Abstract

**Aim:**

To assess the efficacy of the multidisciplinary care (MDC) model for patients with chronic kidney disease (CKD).

**Background:**

The MDC model has been used in clinical practice for years, but the effectiveness of the MDC model for patients with CKD remains controversial.

**Methods:**

Embase, PubMed, Medline, the Cochrane Library, and China National Knowledge Infrastructure databases were used to search for relevant articles. Only randomized controlled trials and cohort studies were pooled. Two independent authors assessed all articles and extracted the data. The efficacy was estimated from the odds ratios and corresponding 95% confidence intervals. A random effects model was used according to the heterogeneity.

**Results:**

Twenty-one studies including 10,284 participants were analyzed. Compared with the non-MDC group, MDC was associated with a lower risk of all-cause mortality and lower hospitalization rates for patients with CKD. In addition, MDC also resulted in a slower eGFR decline and reduced temporary catheterization for patients receiving dialysis. However, according to the subgroup analysis, the lower rates of all-cause mortality in the MDC group were observed only in patients in stage 4–5 and when the staff of the MDC consisted of nephrologists, nurse specialists and professionals from other fields. The most prominent effect of reducing the hospitalization rates was also observed in patients with stage 4–5 but not in patients with stage 4–5 CKD.

**Conclusions:**

MDC can lower the all-cause mortality of patients with CKD, reduce temporary catheterization for patients receiving dialysis, decrease the hospitalization rate, and slow the eGFR decline. Moreover, the reduction in all-cause mortality crucially depends on the professionals comprising the MDC staff and the stage of CKD in patients. In addition, the CKD stage influences the hospitalization rates.

## Introduction

Chronic kidney disease (CKD) is defined as 3 or more months of either kidney damage or an estimated glomerular filtration rate (eGFR) < 60 ml/min/1.73 m^2^ [1]. According to published data from developed countries, the prevalence of CKD has reached 10–13% in the general population [2]. A simulation model forecasts that the prevalence of CKD in adults aged 30 or older is likely to increase from the current value of 13.2–14.4% in 2020 and 16.7% in 2030 [3].

Many risk factors cause CKD progression, such as hypertension, diabetes, old age, and nephrotoxic drugs, resulting in the progression of kidney dysfunction and leading to end-stage renal disease (ESRD), which requires renal replacement therapy (RRT) [4, 5]. According to the annual report of the Bureau of National Health Insurance (BNHI) in Taiwan, patients with ESRD in Taiwan accounted for 0.23% of the local population, but they accounted for 7.2% of the healthcare costs [6]. Due to its epidemiologic features, increasing mortality and substantial healthcare costs, CKD is an internationally recognized public health problem [7].

Because of the prevalence and severity of CKD, guidelines recommend a shift from regarding kidney disease as a life-threatening disorder that requires nephrology referral to a common disorder of varying severity that not only calls for the attention of general internists but also a concerted public health approach for prevention, early detection, and management [8–10]. The ultimate goal of CKD management is to delay disease progression, minimize complications, and improve quality of life. The multidisciplinary care (MDC) model has become a choice in clinical practice to achieve this goal. The MDC model is an integrative medical care system that encompasses a range of disciplines with different but complementary skills, knowledge, and experience to improve health care and achieve the best possible outcomes to meet both the physical and psychosocial needs of patients [11].

The MDC model has been applied in clinical settings for patients with heart failure, patients with cancer, etc. [12, 13]. The application of MDC in patients with CKD has also attracted an increasing attention, although the outcome of MDC for patients with CKD is still controversial, particularly regarding all-cause mortality, temporary catheterization for dialysis, and hospitalization rates [6, 14–20]. MDC has been shown to decrease the all-cause mortality of patients with CKD, reduce the need for temporary catheterization for patients requiring dialysis, and decrease the hospitalization rate [6, 14, 15, 21]. However, other studies have not identified significant differences in these aspects between MDC and non-MDC groups [19, 20, 22].

Based on these findings, we noticed that the composition of MDC staff and the time of referral of patients with CKD to MDC varied. According to published articles, the staff comprising MDC teams vary from a team consisting only of a nephrologist and nurses to teams consisting of a nephrologist, nurses, pharmacists, dieticians, and other professionals [19–23]. Consequently, the questions of whether the composition of the MDC staff influences the therapeutic outcomes of CKD and, if so, whether only a nephrologist and nurses are sufficient to achieve the target have not been answered. However, some patients with CKD are referred to MDC at earlier CKD stages, 1–3, whereas others are referred to MDC at stage 4–5 [15–19]. The questions of whether the time to referral to MDC will influence the outcomes of CKD and, if so, whether the outcomes improve if patients with CKD are referred to an MDC group at an earlier stage remain controversial. Thus, our study also analyzes the influence of MDC staff and CKD stages on the MDC of patients with CKD in published studies.

## Methods

### Search strategy

We searched Embase, PubMed, Medline, the Cochrane Library, and China National Knowledge Infrastructure (CNKI) databases to identify the outcomes of the MDC model for patients with CKD through Aug. 2016. The keywords used for the search were: “multidisciplinary care,” “interdisciplinary care,” “team-based care,” “collaborative project,” “team project,” “chronic kidney disease,” “end-stage renal disease,” “chronic renal failure,” “mortality,” “hospitalization,” “estimated glomerular filtration rate (eGFR),” and “temporal catheterization.” Our search did not have a language restriction; we also manually searched the references of selected articles to identify additional potentially relevant studies.

### Selection criteria

The studies that met the following criteria were included: (1) articles published in English or Chinese journals from Jan. 1990 to Apr. 2016; (2) studies that reported MDC for adult patients with CKD; and (3) MDC was defined as follows: ① The staff of the MDC group comprised at least nephrologists and nurses. ② For the operation model of the MDC, patients with CKD were managed and educated with medical management and lifestyle modifications according to the different stages of CKD. In addition, (4) studies were restricted to randomized controlled trials (RCTs) or cohort studies, and (5) outcomes related to MDC for patients with CKD concentrated on all-cause mortality, hospitalization, temporal catheterization, and eGFR.

### Data extraction

Two investigators (HJ and SY) independently reviewed the included publications and extracted the data using a standardized data collection form. The extracted data included the first author’s name, year of publication, study design, patients’ mean age, sample size, number of participants in each group, duration of study, country of origin, stages of CKD, the composition of the MDC team, all-cause mortality, hospitalization, temporal catheterization for dialysis, and eGFR. Any inconsistency was resolved by discussion or a third author (JCX).

### Assessment of the risk of bias in the included studies

The following items were independently assessed by two authors using the risk of bias assessment tool (Higgins 2011): (1) random sequence generation; (2) allocation concealment; (3) blinding of participants and personnel; (4) blinding of outcome assessment; (5) incomplete outcome data; (6) selective outcome reporting; and (7) other forms of bias (e.g., industry funding).

### Statistical analysis

The Review Manager 5.3.2 software was applied to perform all meta-analyses. We calculated odds ratios (ORs) and 95% confidence intervals (CI) for each study. A random effects model or fixed-effects model was used according to the level of heterogeneity. If considerable heterogeneity was present, a random effects model was utilized; otherwise, the fixed-effects model was used when *I* ^2^ was less than 50% and the Cochran *Q* value had a *p* value ≤0.05.

## Results

### Literature search

**Fig. 1:**
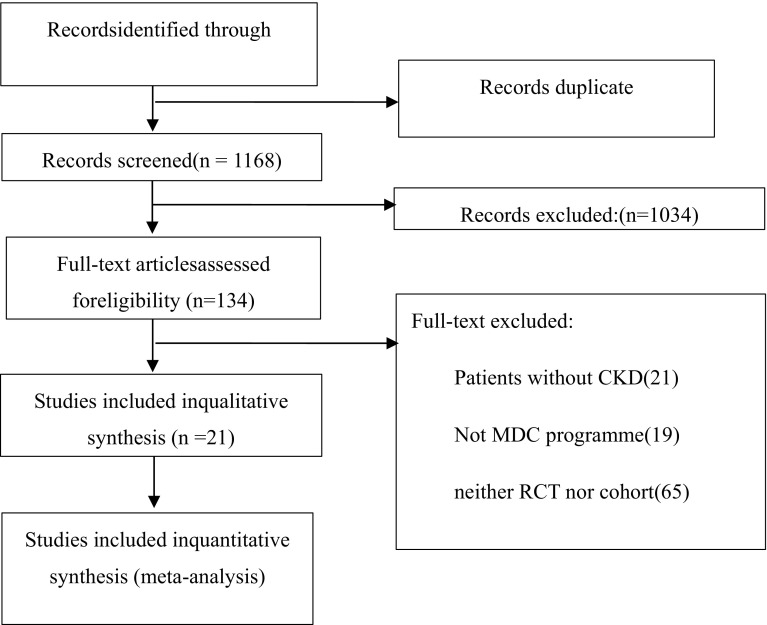
Flowchart of meta-analysis

Figure 1 shows the flowchart. A total of 7556 records were pooled in our study, and 6388 articles were rejected because those articles occurred repeatedly in different databases. A total of 1034 records were removed at the level of title or abstract for articles that had little obvious relevance to our study aims. One hundred thirty-three full-text articles were assessed for eligibility, and 113 full-text articles were excluded for the following reasons: 21 studies did not examine patients with CKD; 19 studies did not involve MDC; 65 studies were neither RCTs nor cohort studies; 7 studies were excluded due to the absence of a description of the OR, and 1 study was excluded because it used the same participants as another article. Finally, 21 studies were included in our study.

### Study characteristics

Within the selected 21 studies, 5 studies were RCTs, and 16 studies were observational cohort studies. The basic characteristics of the included studies are listed in Table 1. Three studies were performed in patients with stage 3B-5 CKD; one study was performed using patients with stage 1–5 CKD; one study was performed using patients with stage 3–4 CKD; 7 studies were performed using patients with stage 3–5 CKD; 4 studies were performed using patients with stage 4–5 CKD; 4 studies were performed using patients with stage 5 CKD; and one study was performed using patients with stage 2–5 CKD. The mean duration of follow-up was 3.02 ± 1.44 years (1–5.7 years). Six studies were performed in Taiwan; 6 studies were performed in Canada; and 3 studies were performed in the USA. One study each was performed in China, the UK, France, Hong Kong, Korea, and the Netherlands. In all 21 studies, nurses and nephrologists were included in the MDC team. A dietician was included in 14 studies, a pharmacist in 7 studies, and a social worker in 11 studies.

**Table 1:** The basic characteristics of included studies

References	Design	Age mean	Subjects *N*(*n*1 + *n*2)	Year	Location	Population	MDC component
Barrett [19]	RCT	67	474 (238 + 236)	1.7	Canada	CKD3–4	1 + 2
Chen [14]	Cohort	63	822 (391 + 431)	5	Taiwan	CKD3–5	1 + 2 + 3 + 4 + 5
Chen [21]	Cohort	62	1206 (592 + 614)	3	Taiwan	CKD3B–5	1 + 2 + 3 + 4
Chen [6]	Cohort	65	1056 (528 + 528)	3	Taiwan	CKD3–5	1 + 2 + 3 + 4 + 5
Curtis [16]	Cohort	62	288 (132 + 156)	3.4	Canada, Italy	CKD5	1 + 2 + 3 + 4 + 5
Fenton [17]	Cohort	63	365 (171 + 194)	4	UK	CKD4–5	1 + 2 + 3 + 5
Goldstein [32]	Cohort	58	87 (61 + 26)	2.3	Canada	CKD3–5	1 + 2 + 3 + 4 + 5
Hemmelgarn [33]	Cohort	76	374 (187 + 187)	3.5	Canada	CKD3–5	1 + 2 + 3 + 5
Peeters [22]	RCT	59	788 (395 + 393)	5.7	Netherland	CKD1–5	1 + 2
Rognant [20]	Cohort	66	160 (40 + 120)	1	France	CKD4–5	1 + 2
Wei [18]	Cohort	60	140 (71 + 69)	1	Taiwan	CKD4–5	1 + 2 + 3
Wu [15]	Cohort	63	573 (287 + 286)	1	Taiwan	CKD4–5	1 + 2 + 3
Yeoh [34]	Cohort	60.3	103 (68 + 35)	4	USA	CKD5	1 + 2
Yu [31]	RCT	64	445 (232 + 213)	2.8	Taiwan	CKD3–5	1 + 2 + 3
Chan [23]	RCT	50	205 (104 + 101)	2	Hong Kong	CKD3–5	1 + 2 + 3
Cho [28]	Cohort	58	298 (149 + 149)	3.5	Korea	CKD3B–5	1 + 2 + 3 + 4 + 5
Harris [35]	Cohort	69	437 (206 + 231)	5	USA	CKD3–5	1 + 2 + 5
Devins [36]	RCT	59	297 (149 + 148)	1	Canada	CKD3B–5	1 + 2 + 5
Levin [37]	Cohort		76 (37 + 39)	3.5	Canada	CKD5	1 + 2
Bayliss [38]	Cohort	68	2002 (233 + 1769)	4	USA	CKD2–5	1 + 2 + 3 + 4 + 5
Zhang [39]	Cohort	58	88 (29 + 59)	3	China	CKD5	1 + 2 + 3 + 5

Professionals in MDC include: 1, nephrologist; 2, nurses; 3, dietitian; 4, pharmacists; 5, social workers

*RCT* randomized controlled trial

### MDC and outcomes

**Fig. 2:**
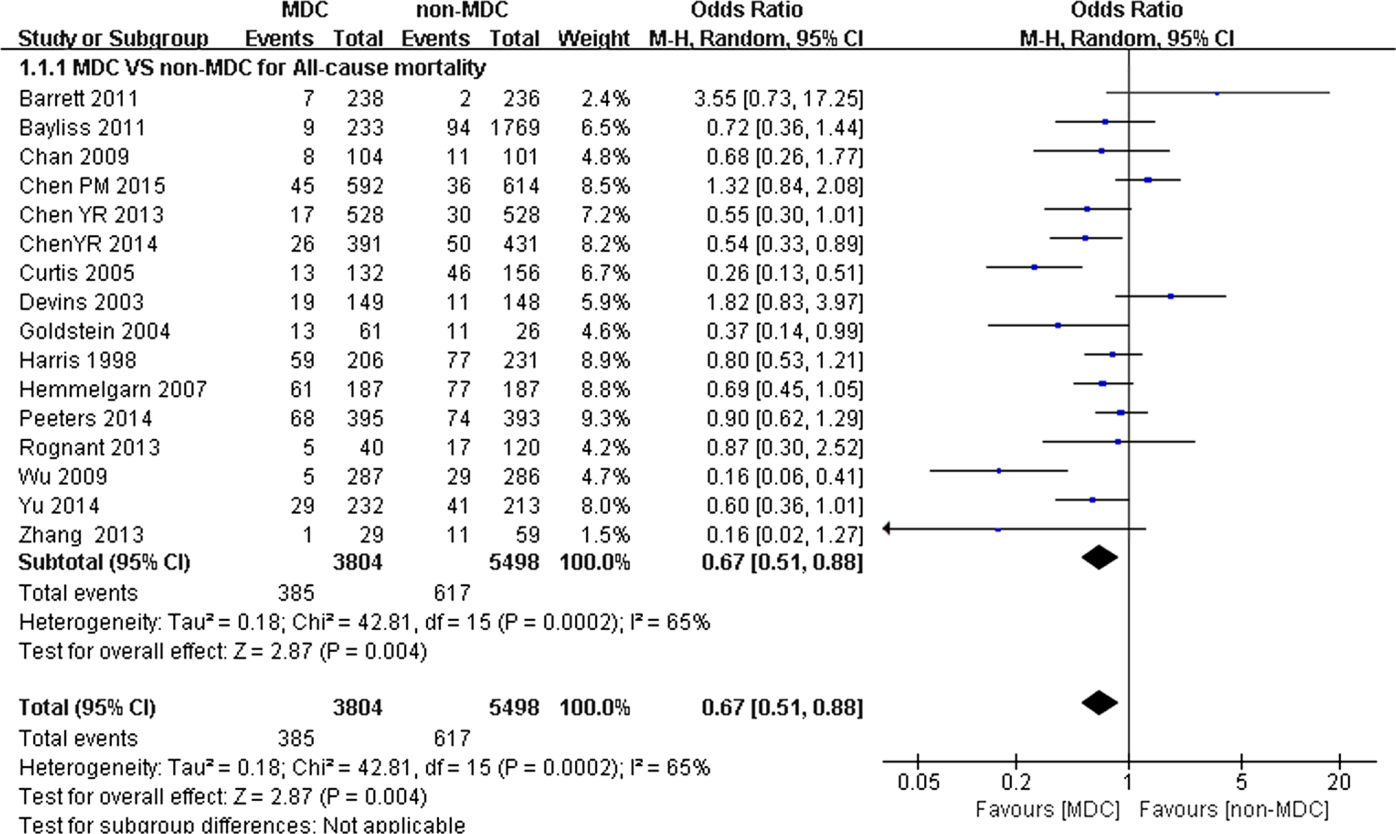
All-cause mortality of chronic kidney disease (CKD) patients on multidisciplinary care (MDC) and on non-MDC

A total of 10,284 participants (4300 participants in the MDC group and 5984 participants in the non-MDC group) with a mean age of 62.75 ± 5.41 years were analyzed in the 21 studies. Among these studies, 16 trials reported the association between MDC and all-cause mortality, revealing that patients with CKD who received MDC had lower all-cause mortality than the non-MDC group. The OR of overall mortality was 0.67 (95% CI 0.51–0.88, *p* < 0.01) (Fig. 2). A random effects model was used because of the heterogeneity between studies (*Q* = 42.81, *I* ^2^ = 65%, *p* < 0.01) (Table 2).

**Table 2:**
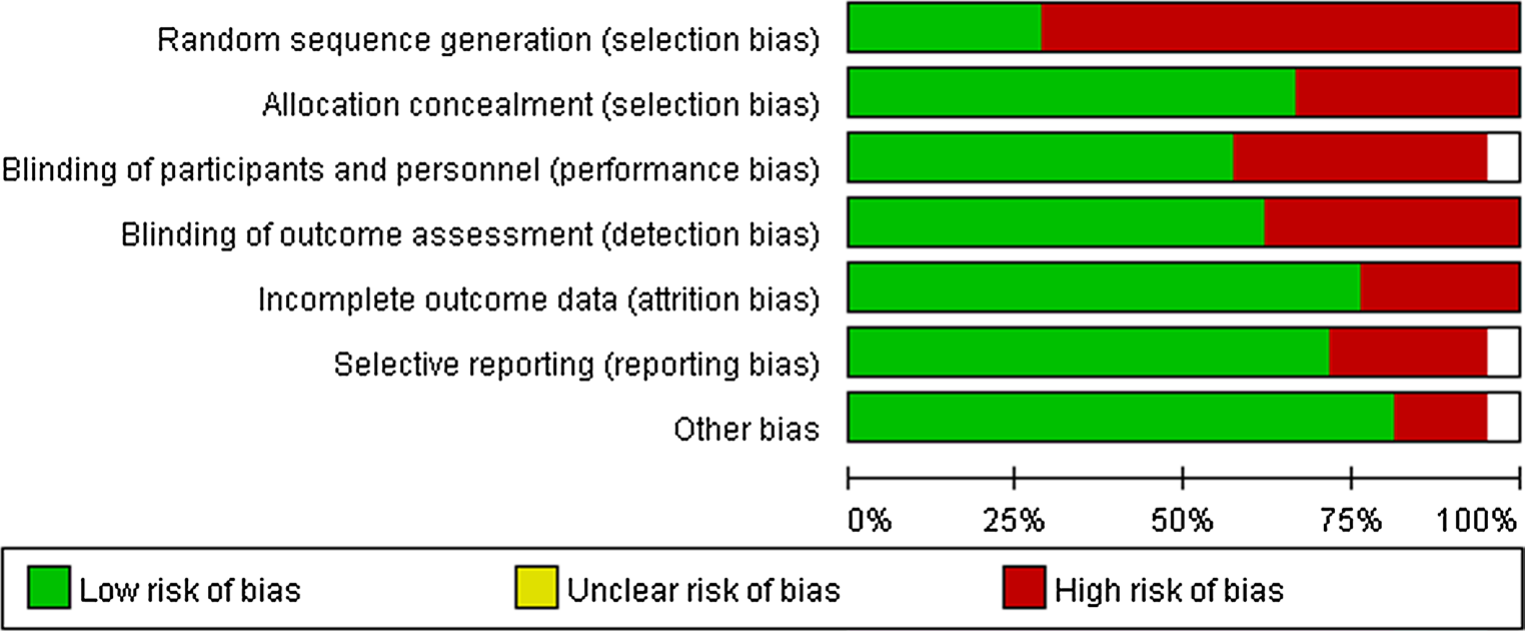
Quality of included studies

**Fig. 3:**
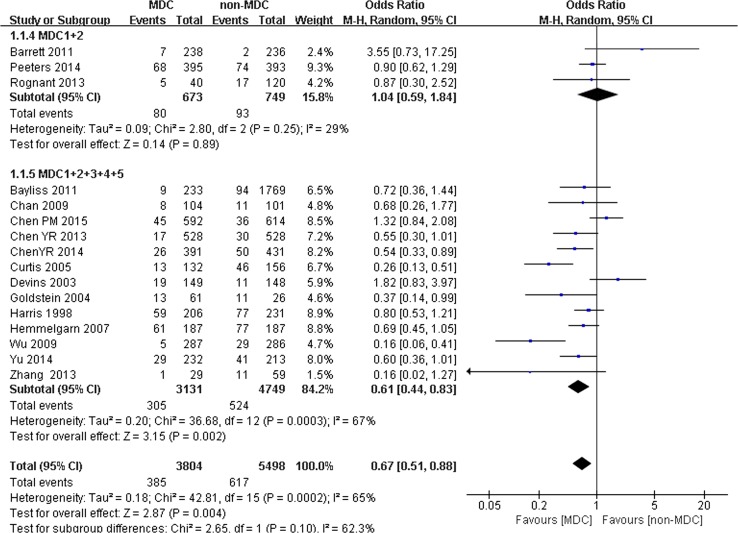
Subgroup analysis of all-cause mortality of chronic kidney disease (CKD) patients on different MDC components

Comparison of all-cause mortality in MDC and non-MDC groups based on differences in the composition of the MDC team:

We performed an analysis to determine whether the composition of the MDC group would influence the all-cause mortality of patients with CKD. Sixteen studies were split into 2 subgroups. One subgroup included 3 studies with 1422 participants (673 participants in the MDC group and 749 participants in the non-MDC group); the MDC team included only nephrologists and nurse specialists. Another subgroup included 13 studies with 7880 participants (3131 participants in the MDC group and 4749 participants in the non-MDC group), in which the members of the MDC team were nephrologists, specific nurses, and professionals from other disciplines. Significant differences in all-cause mortality were observed between the 2 groups. When the membership of the MDC team comprised only nephrologists and specific nurses, no significant difference in all-cause mortality was observed between the MDC and non-MDC groups (OR 1.04, 95% CI 0.59–1.84, *p* > 0.05) (Fig. 3). However, when the membership of the MDC team comprised nephrologists, specific nurses, and professionals from other disciplines (dieticians, pharmacists, or social workers), MDC was associated with a lower risk of all-cause mortality (OR 0.61, 95% CI 0.44–0.83, *p* < 0.01) (Fig. 3).

**Fig. 4:**
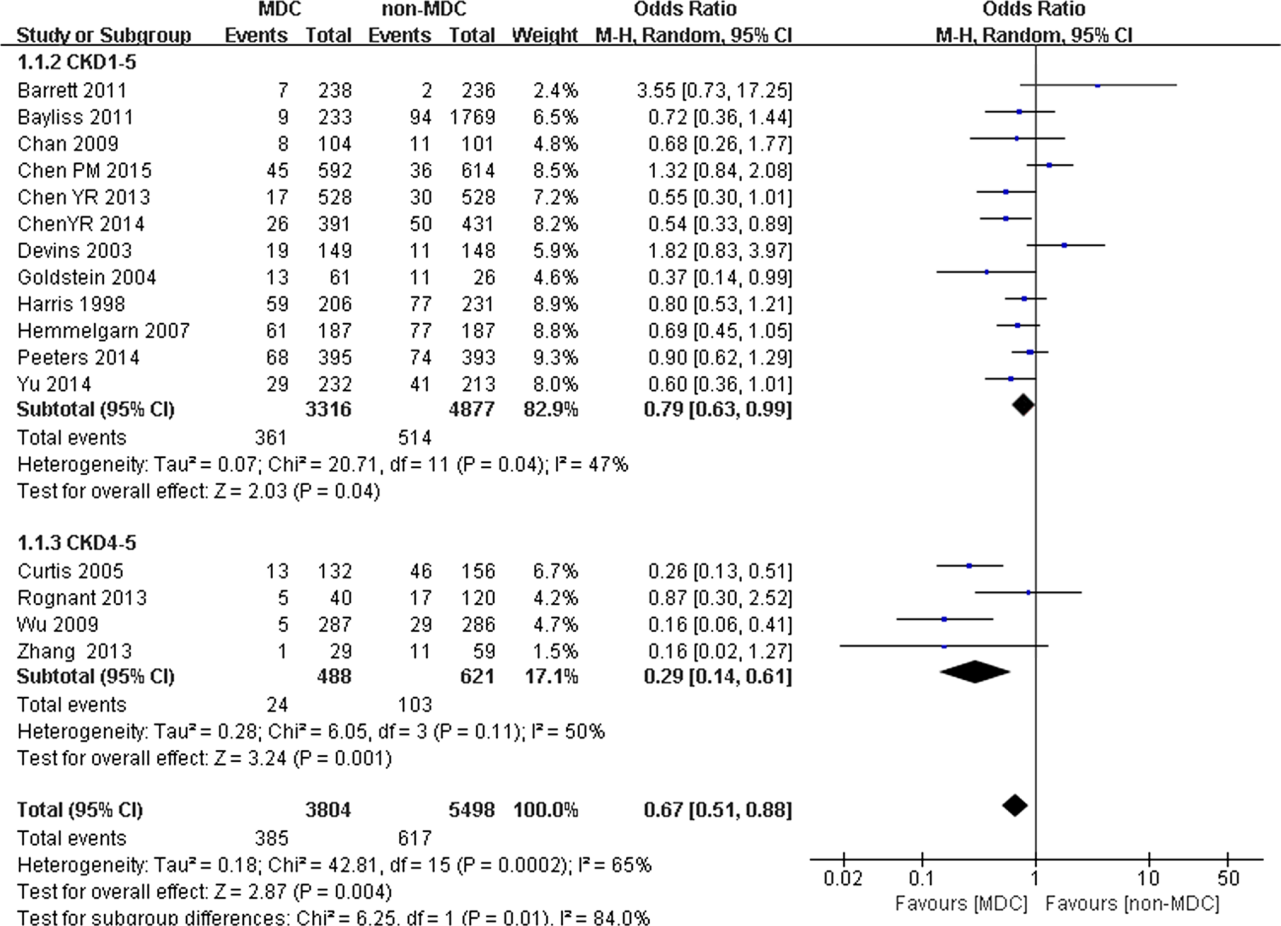
Subgroup analysis of all-cause mortality of chronic kidney disease (CKD) patients on CKD 1–5 and CKD 4–5

Comparison of all-cause mortality between the MDC and non-MDC groups based on different CKD stages:

We performed another study to determine whether different CKD stages would influence the all-cause mortality of patients with CKD. Sixteen studies were split into 2 subgroups. One subgroup included 12 studies with 8193 participants (3316 participants in the MDC group and 4877 participants in the non-MDC group) with stage 1–5 CKD. Another subgroup included 4 studies with 1109 participants (488 participants in the MDC group and 621 participants in the non-MDC group) with stage 4–5 CKD. For patients with stage 1–5 CKD, a slight but significant difference in all-cause mortality was observed between the MDC and non-MDC groups (OR 0.79, 95% CI 0.63–0.99, *p* < 0.05) (Fig. 4). For patients with stage 4–5 CKD, however, significant differences in all-cause mortality were observed between the MDC and non-MDC groups. MDC was associated with a lower risk of all-cause mortality (OR 0.29, 95% CI 0.14–0.61, *p* < 0.01) (Fig. 4).

**Fig. 5:**
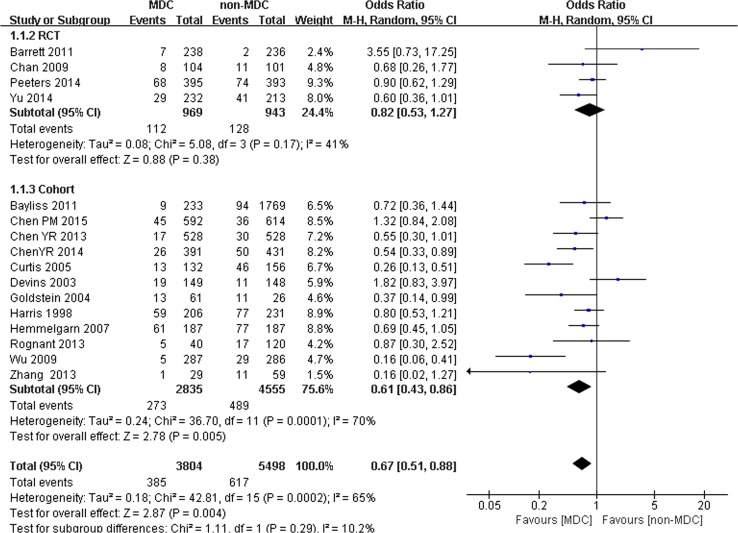
Subgroup analysis of all-cause mortality of chronic kidney disease (CKD) patients on RCTs and cohort

Comparison of all-cause mortality between the MDC and non-MDC groups based on different research techniques:

The lower mortality in patients who received MDC was mainly observed in cohort studies, not in the 4 RCTs. Sixteen studies were split into 2 subgroups. One subgroup included 4 RCTs with 1912 participants (969 participants in the MDC group and 943 participants in the non-MDC group). Another subgroup included 12 cohort studies with 7390 participants (2835 participants in the MDC group and 4555 participants in the non-MDC group). In the RCTs, no significant difference in all-cause mortality was observed between the MDC and non-MDC groups (OR 0.82, 95% CI 0.53–1.27, *p* = 0.38) (Fig. 5). In the cohort studies, significant differences in all-cause mortality were observed between the MDC and non-MDC groups. MDC was associated with a lower risk of all-cause mortality (OR 0.61, 95% CI 0.43–0.86, *p* < 0.01) (Fig. 5).

**Fig. 6:**
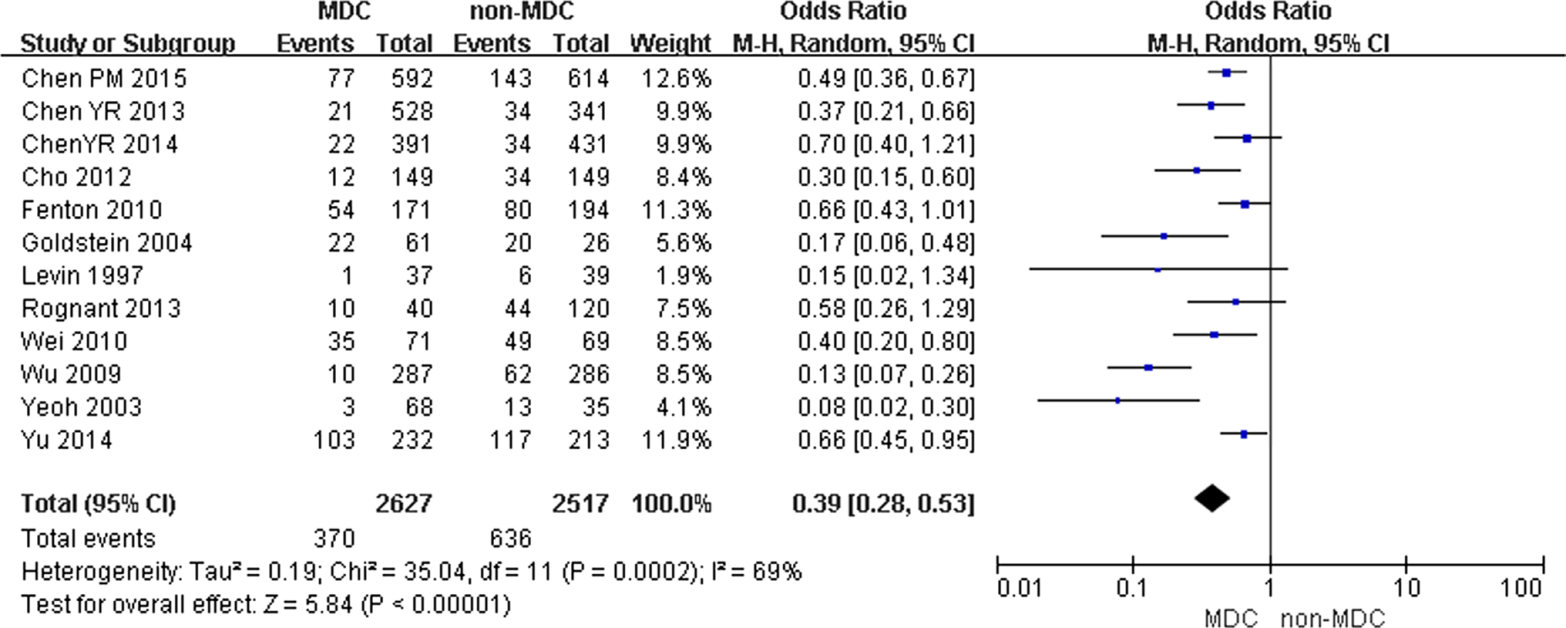
Incidence of temporal catheterization for dialysis in chronic kidney disease (CKD) patients on multidisciplinary care (MDC) and non-MDC

Comparison of temporal catheterization in the MDC and non-MDC groups:

Twelve studies including 5144 participants (2627 participants in the experimental group and 2517 participants in the control groups) examined temporal catheterization for dialysis. MDC was associated with lower risks of temporal catheterization for dialysis, with an OR of 0.39 (95% CI 0.28–0.53, *p* < 0.001). A random effects model was selected because of the heterogeneity between studies (*Q* = 35.04, *I* ^2^ = 69%, *p* < 0.001) (Fig. 6).

**Fig. 7:**
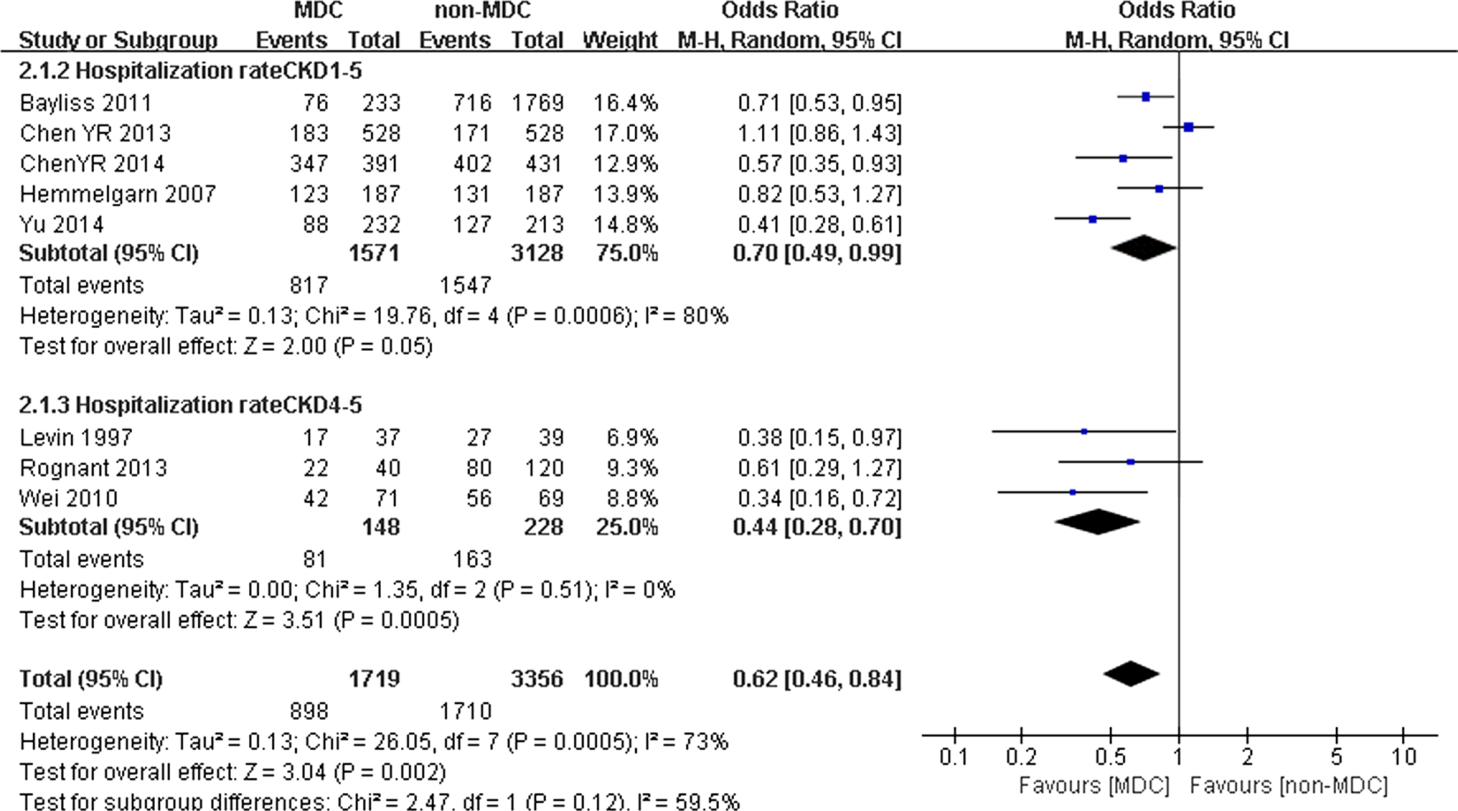
Subgroup analysis of risk of hospitalization for chronic kidney disease (CKD) patients on multidisciplinary care (MDC) and non-MDC

Comparison of hospitalization between the MDC and non-MDC groups:

Eight studies with 5075 participants (1719 participants in the experimental group and 3356 participants in the control group) mentioned hospitalization rates. MDC was associated with a lower hospitalization rate for patients with CKD, with an OR of 0.62 (95% CI 0.46–0.84, *p* < 0.001). A random effects model was selected because of the heterogeneity between studies (*Q* = 26.05, *I* ^2^ = 73%, *p* < 0.001) (Fig. 7). In addition, we performed another analysis to determine whether the different CKD stages would influence the hospitalization rate of patients with CKD. Eight studies were split into 2 subgroups. One subgroup included 5 studies with 4645 participants (1517 participants in the MDC group and 3128 participants in the non-MDC group) with stage 1–5 CKD. Another subgroup included 3 studies with 376 participants (148 participants in the MDC group and 228 participants in the non-MDC group) who were restricted to stage 4–5 CKD. In patients with stage 1–5 CKD, almost no significant difference in hospitalization rates was observed between the MDC and non-MDC groups (OR 0.70, 95% CI 0.49–0.99, *p* = 0.05) (Fig. 7). When patients had stage 4–5 CKD, a significant difference in hospitalization rates was observed between the MDC and non-MDC groups. MDC was associated with a lower risk of hospitalization (OR 0.44, 95% CI 0.28–0.70, *p* < 0.01) (Fig. 7).

**Fig. 8:**
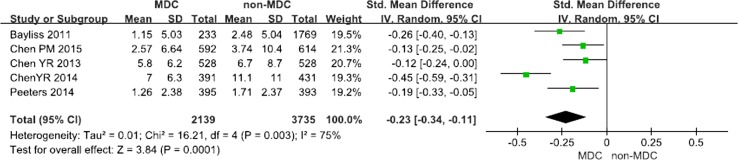
Risk of eGFR for chronic kidney disease (CKD) patients on multidisciplinary care (MDC) and non-MDC

Comparison of the eGFR decline in the MDC and non-MDC groups:

Five articles including 5874 participants (2139 participants in the experimental group and 3735 participants in the control group) examined eGFR decline. MDC was associated with a slower eGFR decline for patients with CKD, with an OR of −0.23 (95% CI −0.34 to −0.11, *p* < 0.001). A random effects model was selected because of the heterogeneity between studies (*Q* = 16.21, *I* ^2^ = 75%, *p* < 0.001) (Fig. 8).

### Publication bias

**Fig. 9:**
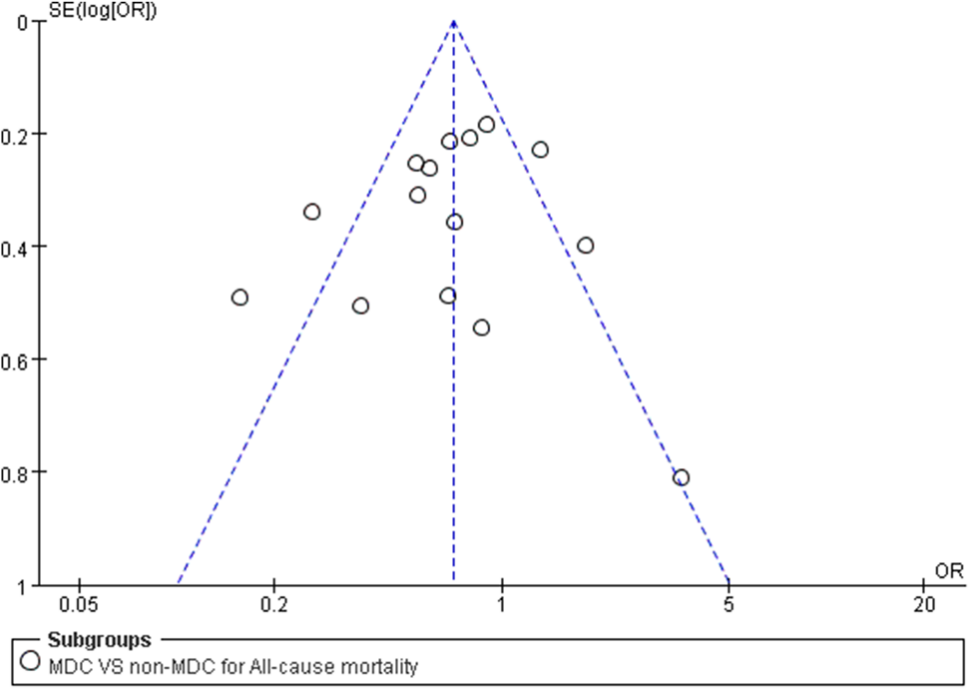
Funnel plot used for exploring the source of publication bias

We did not find evidence of publication bias, based on the results of a funnel plot analysis (Fig. 9); the included studies exhibited a normal distribution.

## Discussion

This systematic review includes 21 studies with a total of 10,284 individuals. The findings of this meta-analysis provide some evidence that MDC might decrease the all-cause mortality of patients with CKD. According to the results of the subgroup analysis, the significant reduction in all-cause mortality was observed only in patients with stage 4–5 CKD, and the staff comprising the MDC teams should include nephrologists, nurses, and professionals from other fields. Additionally, MDC can lower the risk of temporal catheterization for dialysis, slow the eGFR decline, and decrease hospitalization rates, particularly for patients with stage 4–5 CKD.

MDC is patient-centered and integrates independent disciplines to comprehensively manage patients [24]. The integration of the joint efforts of MDC staff is of great importance in helping patients make new adjustments in their lifestyles and efficiently achieve the medication goals formulated in the guidelines. For example, MDC staff make great efforts to decrease the temporary catheterization rate by providing pre-dialysis education, which helps patients fully prepare for dialysis and long-term vascular access, such as an arteriovenous fistula [25–27]. The lower temporary catheterization rates result in a lower risk of infection and cardiovascular disease and lower medical costs [28, 29]. Additionally, the MDC model helps patients strictly control their diets, improves dietary compliance, ensures intensive follow-up to improve patient compliance and adherence, and strengthens patients’ self-care capabilities.

Many factors are associated with the efficacy of MDC, and the clinical interpretation of the efficacy of MDC reported in this meta-analysis should be carefully evaluated. Elements such as the composition and the divisions of the MDC staff and the operation mode of the MDC model were considered. Typically, the staff of MDC models for patients with CKD consists of nurses, dieticians, pharmacists, and social workers in addition to nephrologists [10], all of whom play important roles in the management of these patients. The composition of the MDC staff varied significantly. In some studies, the MDC team included only nephrologists and nurses, but other studies included dieticians and pharmacists as well. Some studies did not identify a significant difference in the all-cause mortality between the MDC and non-MDC groups when the staff of the MDC team included nephrologists and nurses [19, 22]. Other studies, however, revealed significant differences in all-cause mortality between the MDC and non-MDC groups when the staff of the MDC team included nephrologists, nurses, dieticians, and pharmacists [14, 15]. Therefore, the ideal composition of the MDC staff and the extent to which this reduction is clinically meaningful remain uncertain. The evidence from our study suggested a significantly increased risk of all-cause mortality in patients with CKD who were receiving care through an MDC model. However, according to the subgroup analysis, no significant difference in the all-cause mortality was observed between the MDC and non-MDC groups when the staff of MDC is composed of nephrologists and nurses. Lower rates of all-cause mortality in the MDC group existed only when the staff of the MDC team consisted of not only nephrologists and nurses but also professionals from other fields. In other words, the reduction in all-cause mortality vitally depends on the professional staff comprising the MDC team. A possible explanation is that when the MDC staff consists of only nephrologists and nurses, the MDC model is similar to the ordinary model (non-MDC) in which health care is provided by nephrologists and nurses. In fact, when pharmacists and dieticians or other professionals are absent from the MDC group, the education provided to patients with CKD may be insufficient, and thus, patients rarely meet the guidelines for targets such as protein intake limitations, contributing to the deterioration of kidney function. Unfortunately, due to the absence of sufficient data on temporary catheterization rates, hospitalization rates, and declines in eGFR, we were not able to comprehensively assess the risk of those aspects for patients with CKD who were treated using the MDC model in terms of the professionals comprising the MDC staff. We propose that MDC teams that only include nephrologists and nurses may not the best option to consider because the MDC model aims to achieve better outcomes. However, we cannot propose a definitive conclusion on the number of cooperating disciplines that will achieve the best outcomes.

Moreover, the CKD stage was associated with the effectiveness of the MDC model. According to the subgroup analysis, no significant differences in all-cause mortality and hospitalization rates were observed for patients with stage 1–5 CKD. Lower rates of all-cause mortality and hospitalization rates for the MDC group were observed only in patients with stage 4–5 CKD (Fig. 3). A possible explanation is that patients with stage 4–5 CKD tend to have worse kidney function and more complications or morbidity, which might result in a greater risk of death and hospitalization. In addition, the incidence of death occurs over a long period, meaning that the effect of MDC in reducing all-cause mortality is more difficult to observe in patients with earlier stages of CKD in short-term studies that often lasted 1–3 years compared with patients with CKD stage 4–5. In other words, reductions in all-cause mortality and hospitalization rates critically depend on the patients’ stage of CKD. Compared with patients with stage 1–5 CKD, the effect was most prominent in patients with stage 4–5 CKD who received the MDC model. According to our results, the therapeutic outcomes were not improved if patients with CKD were referred to the MDC group at an earlier stage, particularly regarding all-cause mortality and hospitalization rates. In the clinical care of patients with CKD, clinical guidelines recommend that the patients should be referred to nephrologists when they reach stage 4 CKD [30]. Our study also supports this recommendation, although the influence of the CKD stage is not well studied, and further research on the effect of the CKD stage on the MDC model is needed.

However, our study has some limitations and implications that should be considered. First, the CKD stage is not unified, as some studies included stage 1–4, while others included only stage 4–5. Second, the follow-up time varies. Three [19, 23, 31] of the four studies included a follow-up period of <3 years. The effects of MDC on reducing all-cause mortality occur over a long period, and thus, the results from short-term studies must be interpreted with great caution. Finally, we attempted to retrieve all available published and unpublished information, but we cannot exclude the possibility that some published and unpublished studies were overlooked or that published reports might overestimate or underestimate the efficacy of treatments. Moreover, this study included a search of only the Chinese and English literature; reports published in other languages were not included.

In conclusion, MDC may significantly reduce the risk of all-cause mortality, reduce temporary catheterization for dialysis, decrease the hospitalization rate, and slow the eGFR decline. The staff comprising the MDC team and the CKD stage influence the effects of MDC. The significant effectiveness of MDC in reducing all-cause mortality and hospitalization rates was most prominent in patients with stage 4–5 CKD. More studies are needed to confirm our results.
